# Molecular Structure of the Na^+^,K^+^-ATPase α4β1 Isoform in Its Ouabain-Bound Conformation

**DOI:** 10.3390/ijms252212397

**Published:** 2024-11-19

**Authors:** Kazuhiro Abe, Jeff McDermott, Hridya Valia Madapally, Parthiban Marimuthu, Chai C. Gopalasingam, Christoph Gerle, Hideki Shigematsu, Himanshu Khandelia, Gustavo Blanco

**Affiliations:** 1 Department of Chemistry, Faculty of Science, Hokkaido University, Hokkaido 060-0808, Japan; 2 Department of Cell Biology and Physiology, The University of Kansas Medical Center, Kansas City, KS 66103, USA; jmcdermott@kumc.edu; 3 PhyLife: Physical Life Science, Department of Physics Chemistry and Pharmacy, University of Southern Denmark, 5230 Odense, Denmark; hrid@sdu.dk (H.V.M.); hkhandel@sdu.dk (H.K.); 4 Pharmaceutical Science Laboratory (PSL—Pharmacy) and Structural Bioinformatics Laboratory (SBL—Biochemistry), Faculty of Science and Engineering, Åbo Akademi University, FI-20520 Turku, Finland; parthiban.marimuthu@abo.fi; 5 Center for Global Health Research, Saveetha Medical College, Saveetha Institute of Medical and Technical Sciences, Chennai 602 105, India; 6 RIKEN SPring-8 Center, Kouto, Sayo-gun, Hyogo 679-5148, Japan; chai.gopalasingam@riken.jp (C.C.G.); christoph.gerle@riken.jp (C.G.); 7 Japan Synchrotron Radiation Research Institute (JASRI), SPring-8, 1-1-1 Kouto, Sayo, Hyogo 679-5148, Japan; shigematsu@spring8.or.jp

**Keywords:** Na^+^,K^+^-ATPase α4, cryoelectron microscopy, isoform, isozyme

## Abstract

Na^+^,K^+^-ATPase is the active ion transport system that maintains the electrochemical gradients for Na^+^ and K^+^ across the plasma membrane of most animal cells. Na^+^,K^+^-ATPase is constituted by the association of two major subunits, a catalytic α and a glycosylated β subunit, both of which exist as different isoforms (in mammals known as α1, α2, α3, α4, β1, β2 and β3). Na^+^,K^+^-ATPase α and β isoforms assemble in different combinations to produce various isozymes with tissue specific expression and distinct biochemical properties. Na^+^,K^+^-ATPase α4β1 is only found in male germ cells of the testis and is mainly expressed in the sperm flagellum, where it plays a critical role in sperm motility and male fertility. Here, we report the molecular structure of Na^+^,K^+^-ATPase α4β1 at 2.37 Å resolution in the ouabain-bound state and in the presence of beryllium fluoride. Overall, Na^+^,K^+^-ATPase α4 structure exhibits the basic major domains of a P-Type ATPase, resembling Na^+^,K^+^-ATPase α1, but has differences specific to its distinct sequence. Dissimilarities include the site where the inhibitor ouabain binds. Molecular simulations indicate that glycosphingolipids can bind to a putative glycosphingolipid binding site, which could potentially modulate Na^+^,K^+^-ATPase α4 activity. This is the first experimental evidence for the structure of Na^+^,K^+^-ATPase α4β1. These data provide a template that will aid in better understanding the function Na^+^,K^+^-ATPase α4β1 and will be important for the design and development of compounds that can modulate Na^+^,K^+^-ATPase α4 activity for the purpose of improving male fertility or to achieve male contraception.

## 1. Introduction

Sodium potassium adenosine triphosphatase or Na^+^,K^+^-ATPase (EC/7.2.2.13) is a key enzyme and active ion transport system expressed on the surface of most animal cells [1,2]. Na^+^,K^+^-ATPase is a nano machine capable of converting the chemical energy from the hydrolysis of ATP into a transmembrane electrochemical gradient for Na^+^ and K^+^. Translocating those cations in a 3:2 stoichiometry, Na^+^,K^+^-ATPase generates an uneven distribution of Na^+^ and K^+^ across the cell plasma membrane which is vital to many cell processes [3]. Thus, Na^+^,K^+^-ATPase supports cell resting membrane potential, provides cell excitability allowing the production of the cell action potential, and provides the electrochemical force for the Na^+^ coupled co- and counter-transport of different solutes and water in and out of the cell [4,5].

The biochemical events in Na^+^,K^+^-ATPase that lead to ATP hydrolysis and ion translocation consist of a series of reactions known as the Na^+^,K^+^-ATPase reaction cycle. This involves Na^+^-and K^+^-induced structural changes and a shift in the Na^+^,K^+^-ATPase structure between two main states known as the E1 and E2 and a series of intermediate conformations in a cyclic fashion [6,7,8]. These molecular changes are accompanied by the alternating auto phosphorylation and dephosphorylation of the enzyme from ATP and are associated with the binding, occlusion (or trapping), and translocation of Na^+^ and K^+^ across the cell plasma membrane [9,10]. Due to this property of forming a phosphorylated intermediate, Na^+^,K^+^-ATPase is classified as a member of the P-type class of ATPases, which includes a series of other cation and lipid transporting ATPases across the animal kingdom [11,12].

From a structural perspective, Na^+^,K^+^-ATPase is a heterodimer that results from the association of two major proteins, termed the α and β subunits [13,14]. The α or catalytic portion of the enzyme, is a multi-span transmembrane protein of 110–112 kDa with most of its mass, as well as its N- and C-termini facing the inside of the cell. This subunit contains the binding sites for Na^+^, K^+^, ATP, the cardiotonic steroid inhibitor ouabain, and several regulatory proteins that modify Na^+^,K^+^-ATPase function [15]. In addition, fluorinated complexes such as beryllium fluoride (BeF), aluminum fluoride (AlF) and magnesium fluoride (MgF) are known to act as phosphate mimetics of Na^+^,K^+^-ATPase, binding and stabilizing it in the phosphorylated E2 conformation [16]. Like other P-type ATPases, the Na^+^,K^+^-ATPase α subunit contains several domains that are structurally and functionally distinct. These include the following: (1) the transmembrane or transport region that has ten alpha helices and forms the passage through which ions move across the cell membrane; (2) the actuator or A region, a globular domain that contains the signature TGE motif common to all P-type ATPases; (3) the phosphorylation or P domain that harbors the conserved Pi acceptor Aspartate and Mg^2+^ binding sites; and (4) the nucleotide or N domain that binds ATP and phosphorylates the P-domain [17]. The β polypeptide is a 40–60 kDa single membrane-spanning protein, with a large ectodomain that is heavily glycosylated, and the N-terminus is located in the cytosol. The β subunit is required for Na^+^,K^+^-ATPase activity and is important for the folding, stability, and targeting of the whole enzyme to the appropriate domain on the cell surface [18,19].

Na^+^,K^+^-ATPase exists as the product of different genes, which encode for different molecular variants of the α and β subunits. Currently, four α isoforms, named α1, α2, α3, and α4, and three β subunits, termed β1, β2 and β3, have been described in mammals [20,21,22,23,24,25]. Primary structure comparisons show a high degree of conservation for a specific α or β isoform across species, with identity scores between 92 and 96% for the α and 94–96% for the β polypeptides. The amino acid conservation among isoforms is lower, which highlights the biological relevance of the molecular diversity of the Na^+^,K^+^-ATPase. From a structural perspective, Na^+^,K^+^-ATPase α4 is the most divergent Na^+^,K^+^-ATPase α variant, presenting 76–78% amino acid identity compared to the α1, α2, and α3 isoforms [26]. Both α and β proteins are arranged in different combinations depending on the cell type considered and their expression either varies during tissue development or is differentially affected by disease [26]. The Na^+^,K^+^-ATPase α1 and β1 polypeptides are ubiquitously expressed in all tissues, while the other isoforms have a more restricted pattern of expression [20]. Na^+^,K^+^-ATPase α4 has a unique distribution, being confined to the male germ cells of the testis [27,28,29]. Na^+^,K^+^-ATPase α4 is upregulated after the meiotic stage of spermatogenesis to reach its highest levels in the mature spermatozoa, where it mainly localizes to the sperm flagellum [30]. Na^+^,K^+^-ATPase α4 is important for maintaining the low intracellular Na^+^ levels required to sustain sperm membrane potential, cell intracellular Ca^+2^ levels, and the appropriate cell pH [31]. Due to these actions, Na^+^,K^+^-ATPase α4 is essential for sperm motility and hyperactivation, a key event that is associated with sperm capacitation and is required for the sperm ability to fertilize the oocyte [32]. Moreover, when Na^+^,K^+^-ATPase α4 is deleted in mice, only the males are completely infertile due to severe asthenoospermia [33,34]. From a biochemical standpoint, Na^+^,K^+^-ATPase α4 has specific functional properties that distinguish it from the other Na^+^,K^+^-ATPase isoforms. Thus, α4 has a relatively higher apparent affinity for Na^+^, a lower apparent affinity for K^+^, an intermediate affinity for ATP, and a high sensitivity to inhibition by ouabain compared to α1, α2 and α3 [28,30,35,36].

At present, the structure of Na^+^,K^+^-ATPase α1 in both its E1 and E2 states and in the presence of the β1 subunit has been determined for the enzyme from pig and bovine kidney, as well as for the shark rectal glands [37,38,39]. However, less is known regarding the Na^+^,K^+^-ATPase α2 and α3 isoforms and no information is available for the tertiary structure of Na^+^,K^+^-ATPase α4. Due to the critical role that Na^+^,K^+^-ATPase α4 plays in sperm function, our focus has been placed on this protein as an attractive target for the control of male fertility, both to enhance it as a mechanism to improve male fertility, or to block it for the purpose of male contraception [40,41]. The development of pharmacological agents directed to modify Na^+^,K^+^-ATPase α4 function will be highly facilitated by understanding the real structure of this protein, rather than relying on in silico homology models which are less accurate. In this work, we used cryoelectron microscopy (cryo-EM) to decipher the structure of human Na^+^,K^+^-ATPase α4 in combination with the β subunit in its ouabain-binding conformation and in the presence of beryllium fluoride, which corresponds to the E2 phosphate conformation of the protein [42].

## 2. Results and Discussion

### Cryo-EM Structure of Na+,K+-ATPase α4 in the Ouabain-Bound E2P Form

**Figure 1:**
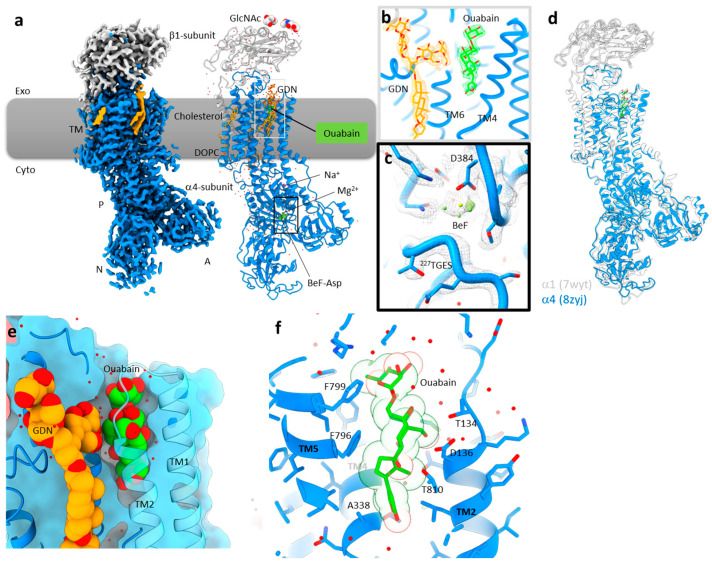
Structure of human Na^+^,K^+^-ATPase α4 with bound ouabain. (**a**) Overall cryo-EM structure of α4 in the ouabain-bound E2P state, viewed from the membrane plane showing the Na+,K+-ATPase α4 (blue) and β subunit (gray). Yellow density and stick models correspond to the detergent (GDN) and lipids (DOPC and cholesterol) present. N-linked glycans are shown as spheres. The membrane position (exoplasmic side-up) is indicated by a gray box. (**b**,**c**). Density map in the region surrounding the ouabain-binding sites (**b**) and in the P domain, where BeF3 binds to the conserved P-type ATPase aspartic acid (**c**). (**d**) Comparison of the α4 model (blue) with α1 in the same conformation (light gray, ouabain-bound E2P, 7wyt). (**e**) Clipped membrane slice of ouabain and GDN bound region at the exoplasmic surface of the membrane. Ouabain (green) and GDN (yellow) are shown as spheres. TM1 and TM2 are shown as transparent helixes for clarity. (**f**) Close-up view of the ouabain-binding site of Na^+^,K^+^-ATPase α4. Transparent sphere shows van der Waals volume of ouabain.

To determine the ouabain-binding pose of the testis specific isoform of Na^+^,K^+^-ATPase, we expressed the human Na^+^,K^+^-ATPase α4β1 isozyme in HEK293S cells in an active form. After isolation by column chromatography, the enzyme was subjected to beryllium fluoride and ouabain. Following cryo-EM analysis, we obtained a structure at 2.37 Å resolution (Figure 1 and Figure S1, Movies S1 and S2). Overall, the molecular structure of Na+,K+-ATPase α4 (Figure 1a) resembled that of Na^+^,K^+^-ATPase α1 under the same conditions [43] (7wyt, Figure 1d) (RMSD = 0.894 Å), showing the typical transmembrane, A, N, and P domains that characterize the P-type ATPase family. The phosphate analog beryllium fluoride (BeF) mimics phosphorylation and is known to lock Na^+^,K^+^-ATPase in a functional state that corresponds to the E2 phosphate conformation of the transporter [42]. In our structure, BeF was found to be bound to the reaction center Asp in the invariant DKTG sequence at the P domain, which is covered by the conserved TGES loop from the A domain (Figure 1c). This region forms a compact headpiece, which is characteristic of the E2P state of Na^+^,K^+^-ATPase α1. Due to the arrangement of the cytoplasmic domain, TM1 to TM4 adopted an exoplasmic-open arrangement to which ouabain binds. The conditions under which we prepared Na^+^,K^+^-ATPase α4 provides insights into the E2P conformation of the protein but does not allow us to make direct correlations with the particular affinity of this isoform for Na^+^ and K^+^. Since the amino acid residues that directly coordinate Na^+^ and K^+^ are conserved in α1 and α4, secondary or tertiary (indirect) interactions around the cation binding sites, or changes in the E1–E2 equilibrium, may explain the different “apparent” affinities for ions that Na^+^,K^+^-ATPase α4 has. Further structural experiments under Na^+^ and K^+^ conditions will be required to ascertain the isoform specific differences in cation binding.

The ouabain-binding mode is unambiguously determined by its clear EM density (Figure 1b, Movies S3 and S4). Close to the ouabain-binding site, we observed another clear non-protein density area, corresponding to a GDN (glycol-diosgenin), detergent molecule, which was used for purification of the protein. The head group glycan of GDN penetrates toward the ouabain-binding site via the side fenestration formed between TM2 and TM6 (Figure 1b,e). The EM densities for two GDN molecules are clearly discriminated from those corresponding to ouabain and cholesterol, by their headgroups facing to the extracellular side (Figure 1b, Movies S1 and S3). We also found one cholesterol molecule bound to the peripheral TM, for which no indication of a sugar moiety even in the low-contoured EM density map can be distinguished (Figure 1a).

**Figure 2:**
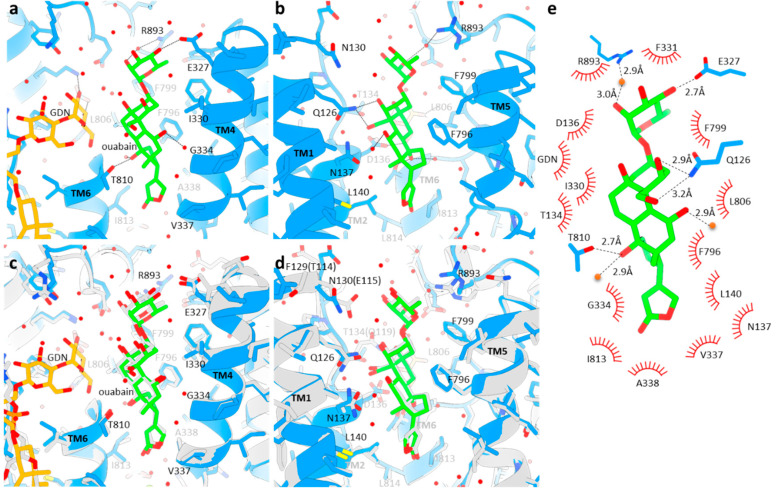
Ouabain-binding site. (**a**,**b**) Molecular details of ouabain-binding viewed from the direction in which TM1 and TM2 are located (**a**) and that of TM3 and TM4 (**b**), parallel to the membrane with exoplasmic side-up. TM1, TM2 or TM3 and TM4 are removed in each figure for clarity. Dotted lines indicate hydrogen bonds. Red spheres show water molecules. (**c**,**d**) Structure of Na^+^,K^+^-ATPase a1 (gray, 7wyt) superimposed to the structures in a and b. Only residues different from Na^+^,K^+^-ATPase a1 and Na^+^,K^+^-ATPase a4 are indicated in parentheses (numbering corresponding to Na^+^,K^+^-ATPase a1). (**e**) Schematic representations of ouabain binding. Residues that are located 3.9 Å from ouabain are shown. Expected hydrogen bonds within 3.3 Å are depicted as dotted lines.

The overall binding mode of ouabain in Na^+^,K^+^-ATPase α4 is similar to that in Na^+^,K^+^-ATPase α1, with differences relating to the specific isoform amino acid sequences (Figure 2). The five-membered lactone ring of ouabain rests facing the Na^+^,K^+^-ATPase cation binding site but does not reach to that site, it only forms van der Waals contacts with hydrophobic residues (Val337, Ala338, Gly324, Ile813), as shown in Figure 2a,b. While Mg^2+^ was present in our preparation, except for a few water molecules, we could not detect this cation as a strong density at the cation-binding site. This is probably due to the low concentration of Mg^2+^ (1 mM) in the sample. The steroid core of ouabain is surrounded by hydrophobic amino acids located at the cation-transporting conduit connecting the exoplasmic solution to the cation-binding site (Figure 2). In particular, the side chain from Phe796 in TM6 smoothly fits the bowing structure of the steroid core (Figure 1f). This tight contact and the presence of multiple hydrophobic interactions exclude water molecules from the vicinity of ouabain, which provides a favorable increase in entropy to the system.

Although ouabain has several hydroxyl groups projecting from its sterol nucleus, only two residues (Gln126, Thr810, conserved in α1) and three water molecules are likely to form hydrogen bonds with them (Figure 2a,b,e). In Na^+^,K^+^-ATPase α1, Gln119 forms a hydrogen bond with a hydroxyl group in the 6-membered ring (ring A) of the steroid structure next to the rhamnose (Figure 2c,d), which is replaced in Na^+^,K^+^-ATPase α4 for the shorter residue Thr134. The rhamnose tail of ouabain is surrounded by several water molecules at the exit site of the exoplasmic conduit, consistent with its hydrophilic property. Hydrophilic amino acids (Glu327, Arg893) also form, via water molecules, hydrogen bonds with the rhamnose hydroxyl, which helps stabilize its binding pose (Figure 2). In Na^+^,K^+^-ATPase α1, Glu115 forms a hydrogen bond with rhamnose, but its conservative replacement in Na^+^,K^+^-ATPase α4 for Asn130 is slightly farther from rhamnose. This may be due to the replacement of Thr114 (in Na+,K+-ATPase α1) to Phe129 (in Na^+^,K^+^-ATPase α4) that changes the chain trace of TM1-2 loop where Gln130 in Na^+^,K^+^-ATPase α4 is located. In summary, while Na+,K+-ATPase α4 establishes less hydrogen bonds with ouabain compared to Na+,K+-ATPase α1, its hydrophobic (entropy-driven) interactions are much stronger. In addition, the binding pocket in Na^+^,K^+^-ATPase α4 for ouabain appears to be smaller, which may explain the tighter binding and higher affinity that Na^+^,K^+^-ATPase α4 has for ouabain [44].

**Figure 3:**
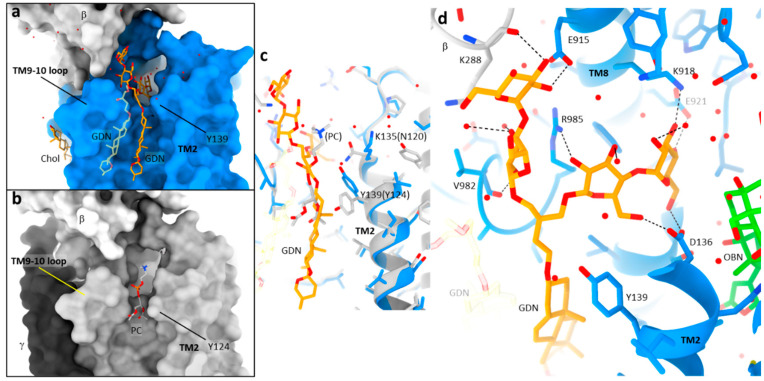
GDN-binding site in Na^+^,K^+^-ATPase α4 (**a**,**b**). Surface representation of Na^+^,K^+^-ATPase α4 (**a**) and α1 ((**b**), pdb: 7wyt) viewed from parallel to the membrane with exoplasmic side-up. Sterol skeleton of GDN (orange sticks) is bound to the longitudinal crevice formed between TM2 and TM9-10 loop, and one of the maltose branches is incorporated to the side fenestration in α4 (**a**), while phosphatidylcholine (PC, gray sticks) is bound to the corresponding position in α1 (**b**). We also modeled another GDN molecule (only the hydrophobic part) and cholesterol (Chol) in the TM peripheral region (**a**). (**c**) Comparison of different rotamer conformation between Na^+^,K^+^-ATPase α1 (gray) and Na^+^,K^+^-ATPase α4 (blue). Amino acids from Na^+^,K^+^-ATPase α1 are indicated in parentheses. (**d**) Close-up view of maltose moieties. Dotted lines connect polar atoms located within 3.5 Å distance.

As mentioned above, we see the detergent GDN as a clear EM density at TM peripherally juxtaposed to the ouabain-binding site (Figure 1b). This indicates that GDN is tightly bound to this position with high occupancy. One of the brunched hydrophilic maltose headgroups of the detergent penetrates in the side fenestration formed between the TM2, TM8 and TM9-10 loop, that continues to the ouabain binding site (Figure 3a,b). Asn120 in Na^+^,K^+^-ATPase α1 is replaced to Lys135 in α4, and their rotamers point to a different direction (Figure 3c). This difference induces altered rotamer conformation of neighboring Tyr139 (Na^+^,K^+^-ATPase α4) and Tyr124 (Na^+^,K^+^-ATPase α1). Consequently, Na^+^,K^+^-ATPase α4 has a proper binding site for GDN at this position as Tyr139 provides a tight fit to GDN, while phosphatidylcholine (PC) is bound in the corresponding position of Na^+^,K^+^-ATPase α1 (Tyr124). Although GDN is an exogenously added detergent and is not present under physiological conditions, it is interesting to speculate that binding of a sugar could be taking place at this position. As several water molecules are visualized, this side fenestration is located at the interface between the outer leaflet of the membrane and the exoplasmic solution. Both maltose branches are coordinated by extensive hydrogen bonds from surrounded hydrophilic residues, including Asp136 from TM2, Glu915, Lys918, Glu921 from TM8, Val982 main chain carbonyl, Arg985 in the TM9-10 loop and the main chain carbonyl group of Lys288 from the b-subunit (Figure 3d). We suspect that a sugar derived from a glycolipid, such as a ganglioside, could interact with Na^+^,K^+^-ATPase α4 in the membrane. Further experiments will be performed to address this potential molecular interaction in the future.

**Figure 4:**
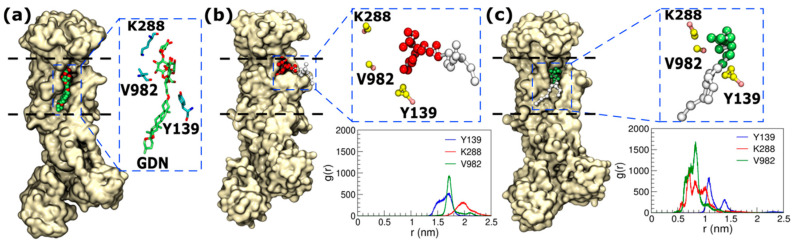
The binding of (**a**) GDN in the cryo-EM structure (**b**) GM1 in the final snapshot of one simulation replica and (**c**) GM3 in the final snapshot of one simulation replica. GM3 binds in a similar configuration in another replica. The binding mode of GM3 is almost identical to GDN. The graphs show the radial distribution functions (histograms of distances) between the lipid head group and the amino acids. The radial distribution functions show that GM3 binds close to residues K288, V982 and Y139. Note that the representation of the amino acids is coarse-grained in (**b**,**c**).

To evaluate the possibility of glycolipids binding to the GDN-binding site, we performed coarse-grained molecular dynamics (MD) simulation of the protein embedded in a model sperm plasma membrane. The composition of the membrane is provided in the methods section. Two sets of simulations were performed, where either GM1 or GM3 was chosen as the representative glycolipid in the membrane. We chose GM1 and GM3 because they are glycolipids known to be present in the sperm membrane. We simulated four replicas of the GM1-system and GM3-system with different initial random distributions of the lipids in the membrane. We find that GM1 binds to the protein in one out of four replicas and GM3 binds to the binding site in two out of four replicas (Figure 4 and Movies S5 and S6). The binding pocket of GM3 is identical to that of GDN as apparent in Figure 4c and the radial distribution functions shown in Figure 4. Although GM1 binds to the same pocket, the mode of binding is different from GDN (Figure 4b). These data indicate that there is a high probability that a glycolipid binds to the protein at the GDN binding pocket, and that the glycolipid is more likely to be GM3 than GM1. It could be possible that the glycolipids regulate enzyme turnover or are necessary for enzyme activation, although further biochemical experiments are required to establish the hypothesis.

## 3. Materials and Methods

### 3.1. Protein Expression and Purification for Structural Analysis

The procedures for protein expression are essentially the same as those reported previously [45,46]. Briefly, Flag epitope tag (DYKDDDDK), a hexa-histidine tag, and the enhanced green fluorescence protein (EGFP) were inserted in the amino terminal side of N-terminal 46 amino acid of the human Na^+^,K^+^-ATPase α4-subunit, followed by a tobacco etch virus (TEV) protease recognition sequence and subcloned into pEZ vector. The wild type human Na^+^,K^+^-ATPase β1-subunit was also cloned. The αβ-complex of Na^+^,K^+^-ATPase were expressed using baculovirus-mediated transduction of mammalian human embryonic kidney (HEK293S GnT1^−^) cells [47], purchased from ATCC.

For cryo-EM analysis, cells were directly solubilized with 1% lauryl maltose neopentyl glycol (LMNG) [48] in the presence of 40 mM MES/Tris (pH 6.5), 10% glycerol, 5 mM dithiothreitol, 1 mM MgCl_2_, in the presence of 1 mM BeSO_4_, 3 mM NaF, 0.1 mM ouabain and protease inhibitor cocktail (Roche; Indianapolis, IN, USA) on ice for 20 min. After removing insoluble material by ultracentrifugation, the supernatant was mixed with anti-GFP nanobody resin [49] at 4 °C for 2 h, which was followed by washing with a buffer containing 40 mM MES/Tris (pH 6.5), 2% glycerol, 1 mM MgCl_2_, 1 mM BeSO_4_, 3 mM NaF, 50 mM NaCl, 0.05 mM ouabain and 0.06% glyco-diosgenin (GDN) [50]. After addition of TEV protease and endoglycosidase, anti-GFP nanobody was incubated at 4 °C overnight. Digested peptide fragments containing EGFP and endoglycosidase were removed by passing the fractions through Ni-NTA resin (Qiagen; Beverly, MA, USA). Flow-through fractions were concentrated and subjected to size-exclusion column chromatography using a Superose6 Increase column (Cytiva, Marlborough, MA, USA) equilibrated in a buffer comprising 20 mM MES/Tris (pH 6.5), 1 mM MgCl_2_, 1mM BeSO_4_, 3 mM NaF, 50 mM NaCl, 0.05 mM ouabain, and 0.06% GDN. Peak fractions were collected and concentrated to 8 mg/mL. A final concentration of 0.1 mM ouabain was added to the protein sample.

### 3.2. ATPase Activity Measurement

Na^+^,K^+^-ATPase α4 was purified in the presence of 300 mM NaCl and in the absence of BeF and ouabain as described above. ATPase activity was determined after diluting the purified enzyme was at 0.05 mg/mL in a buffer containing 40 mM PIPES/Tris (pH 7.0), 300 mM NaCl, 2 mM MgCl_2_, 2 mM ATP di-tris salt in the presence of different concentrations of KCl (0–200 mM). Reactions were initiated by incubating the fractions at 37 °C and maintained for 1 h. Reactions were terminated by the addition of 2 M HCl, and the amount of released inorganic phosphate was determined colorimetrically using a microplate reader (Thermo Fisher, Lenexa, KS, USA) [51]. The specific Na^+^,K^+^-ATPase activity was calculated by subtracting the activities in the absence and presence of 0.1 mM ouabain (Figure S2).

### 3.3. Cryo-EM Analysis

Preparation of the sample and the cryo-EM grids was performed according to a previous report [52]. The purified protein (at 8 mg/mL) containing 0.1 mM ouabain was applied to a freshly glow-discharged Quantifoil holey carbon grids (R1.2/1.3, Cu/Rh, 200 mesh), using a Vitrobot Mark IV (Thermo Fisher, Lenexa, KS, USA) at 4 °C with a blotting time of 4 s under 99% humidity, and then plunge-frozen in liquid ethane. The prepared grids were transferred to a CRYO ARM 300 microscope (JEOL, Boston, MA, USA), operated at 300 kV, with a cold-field emission gun as the electron source, an in-column Omega filter and equipped with a Gatan K3 direct electron detector in the electron counting mode. Imaging was performed at a nominal magnification of 60,000×, corresponding to a calibrated pixel size of 0.753 Å/pix (EM01CT at SPring-8). Each movie was recorded in correlated-double sampling (CDS) electron counting mode for 2.6 s and subdivided into 60 frames. The electron flux was set to 8.46 e^−^/pix/s at the detector, resulting in an accumulated exposure of 60 e^−^/Å^2^ at the specimen. The data were automatically acquired by the image shift method using SerialEM software, version 7.8 [53], with a defocus range of −0.8 to −1.8 μm. The dose-fractionated movies were subjected to beam-induced motion correction, using RELION 3.1 [54], and the contrast transfer function (CTF) parameters were estimated using patch CTF estimation in cryoSPARC (v4, Structura Biotechnology, Toronto, ON, Canada) [55].

For each dataset, particles were initially picked by blob picker using cryoSPARC (v4) and extracted with down-sampling to a pixel size of 3.24 Å/pix. These particles were subjected to several rounds of 2D classifications. Selected classes were then subjected to ab initio reconstruction in three models and refined by non-uniform refinement [55]. The particles from the best class were then re-extracted to the full pixel size and subjected to non-uniform refinement with per-particle defocus refinement, beam-tilt refinement in cryoSPARC (v4). The particle stack was then transferred to RELION 3.1, and subjected to Bayesian polishing [56]. Polished particles were re-imported to cryoSPARC (v4) and subjected to non-uniform refinement. The resolution of the analyzed map was defined according to the FSC = 0.143 criterion [57] (Figure S2, Table S1). The local resolution and angular distributions for each structure were estimated by cryoSPARC (v4). All the models were manually built in Coot [58] using the homology model derived from a crystal structure of ouabain-bound Na^+^,K^+^-ATPase (7wyt) as a template [43]. Phenix (version 20) [59] was used for refinement. The model of Na^+^,K^+^-ATPase with bound ouabain contained 97.6/2.4/0.0% in the favored, allowed, and outlier regions of the Ramachandran plot, respectively.

### 3.4. Molecular Dynamics Simulations

The cryoEM structure of the protein was coarse-grained with elastic network with a force constant of 700 kJ/mol/nm^2^ using Martinize2. The aspartate residue 338ASP was phosphorylated and the phosphorylation was modeled as an extra bead (Qa bead with −2e charge) bound to the aspartate residue [60,61]. The phosphorylated protein was inserted into a bilayer that emulated lipid composition in human spermatozoa using INSANE [62]. The sperm plasma lipid composition used in this study [63] is given in Table 1. Of the lipids in the membrane, 30% had 16:0/18:1 tails and the remaining 70% had 16:0/22:6 tails. Either GM1 or GM3 was used as the glycolipid in the simulations. The Mg^2+^ ion bound to the phosphate group at the phosphorylated aspartate was replaced with a Ca^2+^ ion. The interaction between the Ca^2+^ ion and the phosphate group were retained using a pull code between the two where the distance between them was restrained using a force constant of 1000 kJ/mol·nm^2^ with zero pull rate. Physiological concentration of salt was maintained by addition of Na^+^ and Cl^−^ ions. Martini water beads were used to solvate the system and 10% of them were antifreeze water beads. Four replicas of the system, each with GM1 or GM3 as the glycolipid in the bilayer, were simulated. Energy minimization was performed using the steepest descent algorithm [64] followed by equilibration for 10 ns. The systems were then simulated for 10 μs with an integration timestep of 20 fs. The temperature was maintained at 310 K using V-rescale thermostat [65]. Gromacs v2020.4 [66] was used to perform the simulations. The Martini 2.2 [67] force field was used for the protein while the Martini 2.0 [68] force field was used for the rest of the system.

## 4. Conclusions

While the structure of Na^+^,K^+^-ATPase was obtained for Na^+^,K^+^-ATPase α1 isoform from porcine, bovine, and shark sources [37,38,39], no information was available for the testis-specific Na^+^,K^+^-ATPase α4. In this work, we show for the first time the structure of human Na^+^,K^+^-ATPase α4 in its E2 conformational state, with ouabain bound. Currently, biochemical and functional studies have demonstrated the properties and the essential role that Na^+^,K^+^-ATPase α4 plays in sperm physiology. Data from this work will allow a better understanding of the structure–function relationship of this active ion transporter. Moreover, the Na^+^,K^+^-ATPase structure will aid in advancing the design of compounds that specifically target Na^+^,K^+^-ATPase α4 to modulate its activity for the control of sperm function. This will be important for pharmacological approaches that could be used to improve male fertility in cases of male factor infertility or to inhibit it to achieve male contraception, which has been a long-desired unmet goal. The structure of Na^+^,K^+^-ATPase α4 that we here obtained serves as an improved template, which can be used for a refined compound docking analysis as compared to the in silico homology modeling that was used in the past, or can be applied to deep learning-driven drug design for the de novo synthesis of new chemical scaffolds and improvement in existing compounds [40,69].

## Figures and Tables

**Table 1:** Lipid composition of the bilayer used in this study.

Type of Lipid	Percentage (%)	Inner Leaflet (%)	Outer Leaflet (%)
PC	14.5	31	69
PE	12.35	81	19
Plasmalogen PC	4.9	31	69
Plasmalogen PE	7.84	81	19
PS	3.57	100	0
PI	2.39	100	0
Sphingomyelin	7.34	31	69
Cardiolipin	0.82	50	50
Cholesterol	43.28	46	54
Glycolipid (GM1/GM3)	2.51	0	100

## Data Availability

The structural data generated in this study have been deposited in the Protein Data Bank and EM Data Bank under accession codes 8zyj and EMD-60570.

## Supplementary Materials

The following supporting information can be downloaded at: https://www.mdpi.com/article/10.3390/ijms252212397/s1.
