# Supplementary material for: Molecular Structure of the Na+,K+-ATPase α4β1 Isoform in Its Ouabain-Bound Conformation
Source: Int J Mol Sci. 2024 Nov 19;25(22):12397. doi: 10.3390/ijms252212397 (PMC11594824; doi:10.3390/ijms252212397)
Supplement: Supplementary file 1 [file ijms-25-12397-s001.zip › ijms-3290920-supplementary.pdf]

Supplemental Data

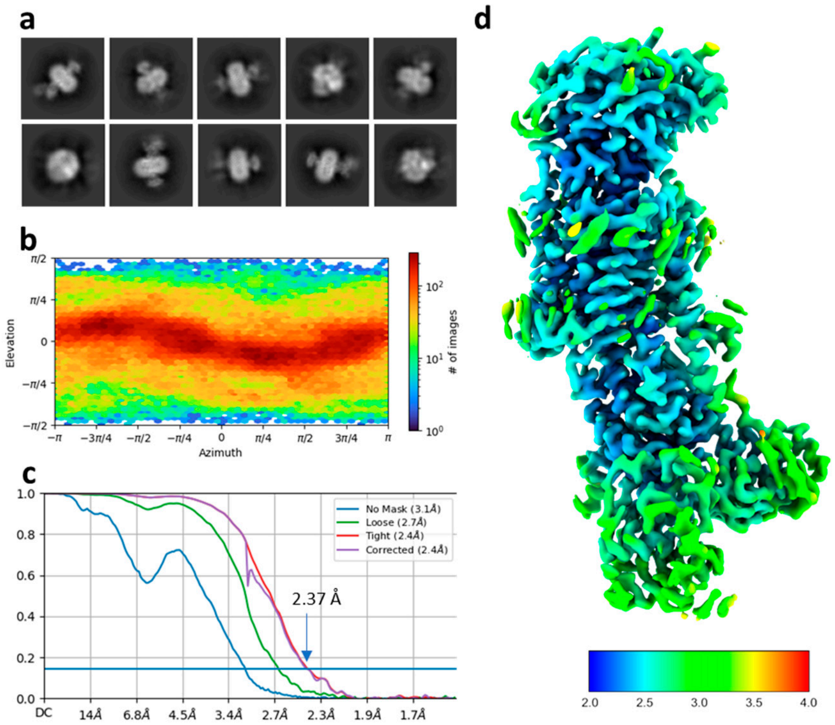

Figure S1. Cryo-EM analysis of Na⁺,K⁺-ATPase α4. (**a**), Representative 2D-class averages. (**b**), Angular distribution plot of particles included in the 3D reconstruction. The number of views at each angular orientation is represented by the color (blue to red). (**c**), FSC plot used for resolution estimation (blue: no mask, green: loose, red: tight, purple: corrected). Dotted line indicates FSC value of 0.143. (**d**), Unsharpened map colored by local resolution as calculated by cryoSPARC (scale is indicated in the figure).

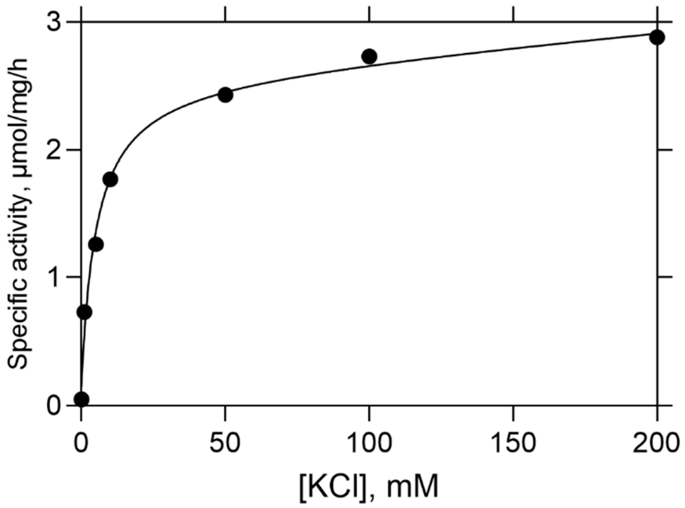

Figure S2. Na⁺,K⁺-ATPase activity of purified human α4β1 complex. Activity was determined by the hydrolysis of ATP as described in "Methods" and using increasing concentrations of KCl as indicated.

Table S1. Statistics for the structural analysis of Na^+,K^+-ATPase α4.

Bound compound	ouabain
PDB ID	8zyj
EMDB	EMD-60570
**Data collection**	
Magnification	60,000
Voltage (kV)	300
Electron exposure (e^-/Å^2)	60
Defocus range (μm)	0.8-1.8
Pixel size (Å/pix)	0.752
Symmetry imposed	*C1*
Movies (no.)	8,303
Initial particles (no.)	3,309,944
Final particles (no.)	162,837
Box size (extract/final, pix)	320/450
Map resolution (Å)	2.37
Map sharpening B-factor (Å^2)	-71.9
FSC threshold	0.143
**Refinement**	
Initial model used (PDB)	7wyt
Model resolution (Å)	2.5
FSC threshold	0.5
Model composition	
Non-hydrogen	10,675
Protein residues	1,274
Waters	302
Ligands	OBN, Na^+, Mg^{2+}, CLR, PCW, 2NAG, 3GDN
B-factor (mean value, Å^2)	
Protein	60.22
Ligand	64.21
Water	55.93
R.m.s. deviations	
Bond length (Å)	0.003
Bond angles (°)	1.056
Validation	
MolProbity score	1.58
Clashscore	4.84
Poor rotamers (%)	1.90
